# Advances in the treatment of traumatic scars with laser, intense pulsed light, radiofrequency, and ultrasound

**DOI:** 10.1186/s41038-018-0141-0

**Published:** 2019-01-29

**Authors:** Xiujun Fu, Jiying Dong, Shen Wang, Min Yan, Min Yao

**Affiliations:** Department of Plastic Surgery, Shanghai Ninth People’s Hospital, Shanghai Jiao Tong University School of Medicine, 639 Zhizaoju Road, Shanghai, 200011 China

**Keywords:** Traumatic scars, Laser, Intense pulsed light, Radiofrequency, Ultrasound

## Abstract

Traumatic scarring is one of the most common complications after soft tissue injury caused by burns and trauma, which affects tens of millions of people worldwide every year. Traumatic scars diminish the quality of life due to disfigurement, symptoms of pain and itch, and restricted motion. The pathogenesis and pathophysiology of traumatic scar remain elusive. The management for traumatic scars is comprised of surgical and non-surgical interventions such as pressure therapy, silicone, corticosteroid, and radiotherapy, which are chosen by clinicians based on the physical examinations of scars. Recently, great progress in treating traumatic scars has been achieved by the development of novel technologies including laser, intense pulsed light (IPL), radiofrequency, and ultrasound. The aim of this review article was to summarize the advances of these technologies for traumatic scars intervention.

## Background

Tens of millions of individuals acquire traumatic scars every year caused by burns and other traumatic injuries worldwide. Scarring is regarded as one of the inevitable consequences of trauma. The prevalence of hypertrophic scar, the most common type of traumatic scars, is reported as high as 70% after burn injury [1]. The traumatic scars especially those on the face and neck are cosmetically unappealing due to dyschromia and irregular texture compared to the surrounding skin. Besides the cosmetic effects, there are several other morbidities associated with traumatic scars. Pruritus and pain associated with traumatic scars are major and very common morbidities. Up to 87% of burn patients especially those with hypertrophic scars report the symptom of pruritus, which disrupts sleep and daily activities [2]. Restricted range of motion of the functional joints and the deformities of facial organs resulted from contracture are among the most severe morbidities. Severe linear or diffuse contracted scars usually require aggressive therapy such as surgical intervention to relieve tension and ultimately improve range of motion and correct the deformities. It should always be noticed that patients with traumatic scars are prone to having anxiety, depression, or even the serious consequence of suicide [3].

Although the complete pathogenesis of traumatic scars needs further elucidated, the formation of pathological scars is regarded as a result of dysregulation in the process of wound healing which characterizes by an inflammatory phase, a proliferative phase, and a remodeling phase [4–6]. The inflammation is crucial to the removal of dead tissue and the prevention of infection by neutrophils and macrophages through the actions of phagocytosis and the secretion of proteases and cytokines. A moderate amount of inflammation is vital to the wound healing process for transition from the inflammatory phase to the proliferative phase. Excessive inflammation response, resulting from infection for instance, often leads to abnormal wound healing and increases the risk of scarring [7]. The proliferation phase was accomplished by the migration and proliferation of various cells. Activated by the cytokines and growth factors, such as transforming growth factor beta (TGF-β) and platelet-derived growth factor (PDGF) released mainly from macrophages, fibroblasts are induced to produce collagen and extracellular matrix. Angiogenesis is initiated by the function of endothelial cells in response to the upregulation of vascular endothelial growth factor (VEGF) [8]. Keratinocytes from the edge of the wound and adnexal structures migrate and proliferate to make the wound healed by re-epithelialization. The remodeling phase can take up to a year or longer to complete, which is characterized by the rearrangement of granulation tissue, the replacement of collagen III by collagen I, and the contracture of the lesion through the action of myofibroblasts. During the remodeling phase, a variety of extracellular matrix (collagen and elastic fiber) and their corresponding enzyme system (matrix metalloproteinases) act to achieve the purpose of restoring normal histological structure [9]. Even after remodeling for many years, the wounded tissue never regains the properties of uninjured skin. Traumatic scars, therefore, are the results of wound healing after tissue injury. The dysregulation of proliferation and apoptosis of fibroblasts, an imbalance between synthesis and degradation collagen in the extracellular matrix, and abnormal structure of epithelium are responsible for scarring [10]. The early stage scars have diffuse capillaries and excessive abnormally arranged collagen fibers in histology, which manifest as red hypertrophic scars, whereas the late stage of scars has closed vessels and excessive fiber deposition, which manifest as normal color or reduced color scars with elevation, flat, or atrophy in morphology [10].

Many treatment options have been developed for the management of traumatic scars, which are divided into surgical and non-surgical approaches [11–13]. Surgical intervention is currently one of the mainstream methods for the treatment of traumatic scars. It is taken to correct the deficiencies and deformities and is especially applicable when a patient has functional impairment caused by contracture of the scar. However, as an invasive technique, surgery has a high risk of inducing the new scar formation and recurrence of scarring. For the treatment of pathological keloid scars, the recurrence rate of surgical excision without adjuvant therapy is as high as 45 to 100% [14]. Non-surgical approaches for the treatment of traumatic scars include compression garments, silicone gel, intralesional therapy with steroids and other medications, radiation therapy, and laser and light therapy. The pressure therapy can improve the scar height by reducing the local blood supply and limiting the oxygen and nutrient for scar tissue, which is mainly used to prevent the further hyperplasia of scar tissue. But the clinical importance is questionable and it cannot effectively treat the scar that has been formed [13, 15]. The silicone products (silicone gel, sheet, strip, cream, spray, or foam) are thought to be capable to effectively inhibit the hyperplasia of scar by multiple mechanisms including hydration, polarization of scar tissue, and elevation of local oxygen tension. However, evidence from studies has yielded contradictory conclusions and it is challenging to come to the definitive conclusion on whether the evidence supports the use of silicone therapy [16, 17]. Local injection of glucocorticoid (triamcinolone acetonide) with or without antineoplastic agents (5-fluorouracil) have been convinced to be effective in certain scar patients in terms of reducing the height and volume of scars, decreasing pain and itch, and making scars more pliable [18]. The side effects of steroids and antitumor agents, however, limit their applications for scar therapy, especially for large scars. Significant benefits are observed with radiation therapy in intractable hypertrophic scar and keloid, but the safety of radiotherapy needs to be evaluated carefully to prevent the secondary radiation carcinogenesis [13, 19].

As traumatic scars are difficult to treat with high recurrence rate, the prevention and treatment of traumatic scars are very challenging for plastic surgeons and dermatologists. Therefore, it is very urgent to develop and explore new treatment options such as laser and light for traumatic scars and optimize the treatment protocols. In recent years, with the rapid development of laser, light, radiofrequency, and ultrasound technologies, clinicians have seen the cosmetic, symptomatic, and functional improvements for the treatment of traumatic scars by these technologies [20]. These new technologies are believed to have the advantages of minimal invasion, fast recovery, and low risk for scar therapy. The current review article aimed to summarize the mechanisms and advances of the treatment of traumatic scars with technologies of laser, intense pulsed light (IPL), radiofrequency, and ultrasound (Table 1).

**Table 1:** Summary of lasers, intense pulsed light, radiofrequency, and ultrasound for traumatic scars

Devices	Mechanism	Clinical outcome
Pulsed dye laser, potassium titanyl phosphate laser, and intense pulsed light	Target hemoglobin and decrease blood supply [25]; increase expression of CTGF [26, 27]	Improve scar color, texture, and pliability [23, 28–31]
Factional lasers	Fractional photothermolysis [33, 34] and collagen remodeling [38–40]	Improve in appearance and contracture [35, 42–44], relieve pain and pruritus [35, 41, 42], and facilitate drug delivery [45, 46]
Radiofrequency devices	Create micro-plasma sparks and induce thermolysis [51–53]	Effective for both hypertrophic and atrophic scars [52, 54] and facilitate drug delivery [57–59]
Ultrasound	Acoustic pressure and “hammering” effect [57]	Adjuvant therapy for traumatic scars [57–59]

*CTGF* connective tissue growth factor

## Review

### Classification of traumatic scars

Traumatic scars are often classified into hypertrophic scar, atrophic scar, flat (superficial) scar, and keloid by clinicians to facilitate clinical management according to their histological and morphological characteristics [21]. They are also categorized into mature and immature scars based on the growth phase, and linear and widespread scars based on the morphology [22]. The characteristics of scars which are critical for the classification include pigmentation, erythema, texture, thickness, and pliability. In the consensus report for laser treatment of traumatic scars published in 2014 by Dr. Anderson et al. [23], traumatic scars were classified taking considerations of scar dyschromia (i.e., erythematous, hyper-pigmented, or hypo-pigmented), scar type (i.e., hypertrophic, flat, or atrophic), body location of the scar (i.e., face, neck, or limbs), and patient characteristics (i.e., skin type and comorbid conditions). This classification aimed to guide the clinicians to choose appropriate lasers and variables for treatment and emphasized that the choice of laser should be concentrated on dyschromia and the relative scar thickness or atrophy as these features are the most prominent appearance features of traumatic scars [23].

## Laser and IPL for the treatment of traumatic scars

Laser and IPL interact with tissue based on the propagation of light through the tissue and the subsequent absorption of photons with conversion to heat, pressure (photoacoustic effect), and photochemical and photobiological reactions. In 1983, Dr. Rox Anderson et al. [24] from Harvard Medical School, firstly introduced the concept of selective photothermolysis of laser and light. With the appropriate wavelength, exposure time, and energy, laser and light are absorbed by hemoglobin, melanin, water, or collagen in skin specifically and the irradiated capillaries, pigment, or scar tissue are selectively affected. Since then, various laser and light devices have been developed and are prevalently used for vascular disease, pigmented disease, hair removal, and scar treatment by dermatologists and plastic surgeons. As the histological characteristics of scars of abnormal microvascular growth and abnormal collagen fibers arrangement have been observed, a variety of laser and light modalities have been developed and taken for both prevention and treatment of traumatic scars mainly based on the principles of selective photothermolysis and fractional photothermolysis.

### Vascular targeted laser or light devices

For the treatment of traumatic scars, pulsed dye laser (PDL), ablative and non-ablative fractional lasers, and IPL are most commonly used laser and light modalities. PDL (585 nm or 595 nm), 532-nm potassium titanyl phosphate laser, and IPL (400–1200 nm; 500–600 nm) target hemoglobin in red blood cells within vessels selectively and achieve the goal to close the local vessels and reduce the blood supply for the growth of scar tissue [25]. To successfully destroy of scar vessels, proper pulse duration which is shorter than the thermal relaxation time of hemoglobin is required. PDL was also indicated to decrease the expression of connective tissue growth factor (CTGF) both in keloid patients [26] and in the in vitro-cultured keloid fibroblasts [27]. CTGF was discovered as strongly profibrotic growth factor via the tissue growth factor/ small mothers against decapentaplegic (TGF/ SMADs) pathway, which is highly expressed in keloid and hypertrophic scars.

PDL and IPL can improve color, texture, and pliability of scars by reducing the pigmentation, vascularity, and bulk of scar tissue [28]. In a clinical study by Manuskiatti et al. [29], 0.45- and 40-ms pulses of 595 nm PDL at the same fluence of 7 J/cm^2^ were compared for the treatment of keloidal and hypertrophic median sternotomy scars. The pulse width of 0.45 ms was proven to be more effective than 40 ms in terms of decreasing scar size and improving scar pliability [29]. PDL treatments at 6-week intervals were integrated into compression therapy in patients from a pediatric burn facility [30]. Less quantitative scar erythema and height and greater tissue elasticity were observed after 2–3 PDL treatments plus compression than with compression alone. PDL given as early as on the day of suture removal to treat the linear operative scars effectively improved the quality and cosmetic appearance [31]. The laser treatment consensus for traumatic scars published in JAMA Dermatology in 2014 [23] believes that if traumatic scars have erythema, the vascular laser (PDL) and light (IPL) devices are initially chosen, and the factional lasers have obvious and synergistic effects with them in the treatment of hypertrophic scars with erythema. Treatment with these devices can be completed without anesthesia, and downtime as well as erythema after treatment is not significant. Cooling is mandated to prevent the side effects of epidermal damage and new scar formation. Special caution is needed when darker-toned scar patients are treated with these devices as the light energy can be competitively absorbed by melanin, which might increase the risk of dyspigmentation [32].

### Fractional lasers

By applying a new concept of skin treatment called fractional photothermolysis, fractional laser creates numerous microscopic thermal injury zones of controlled width (less than 500 μm), depth, and density that are surrounded by a reservoir of spared epidermal and dermal tissue, allowing for rapid repair of laser-induced thermal injury [33, 34]. This technology enables high-energy treatment while minimizing risks of hypo-pigmentation and scarring [35]. Ablative fractional lasers, including 10,600 nm carbon dioxide laser and 2940 nm erbium: yttrium-aluminum-garnet (Er:YAG) laser, use water as the target chromophore and produce columns of vaporized tissue with surrounding eschar and coagulated tissue. The vaporized columns are filled by epidermal cells with complete continuity 48 h post-ablation, and heat shock proteins (Hsp-72 and Hsp-42) are elevated for months [36, 37]. In histology, mature burn scars treated with fractional carbon dioxide laser demonstrated a return toward a fetal collagen profile, with increased type III collagen and decreased type I collagen [38]. The collagen architecture in treated scars was also more closely resembling that of a normal skin [38]. The induction of molecular changes including increased expression of TGF-β3 and matrix metalloproteinase-1 and decreased expression of basic fibroblast growth factor might be accountable for the process of collagen modeling caused by fractional carbon dioxide laser treatment [39]. Non-ablative fractional lasers, e.g., erbium: glass lasers with a wavelength of 1550 nm or 1540 nm, create columns of coagulated tissue composed of denatured collagen while leaving the epidermal layer intact [20]. Scar treated with non-ablative fractional laser shows an interwoven collagen structure with an overall increased similarity to normal unaffected skin post-treatment [40], which was postulated as the subsequence collagen remodeling response induced by columnar coagulation. Although remodeling of collagen induced by fractional lasers has been well accepted, the detailed mechanism underlying this process has yet been fully elucidated.

Currently, a variety of ablative fractional and non-ablative fractional lasers have been developed and they are widely used for the treatment of traumatic scars to improve the appearance and minimize the associated pain and pruritus [35, 41, 42]. The ablative fractional lasers, as well as non-ablative fractional lasers, have been supported to have functional improvement as well for the treatment of scar contracture by significant evidence [35, 42–44]. Combined with surgical scar revision, fractional lasers could induce scar rehabilitation and may eventually decrease the need for scar excision and reduce the morbidity of donor sites [23]. By creating a matrix of microscopic channels penetrating stratum corneum, ablative fractional laser-assisted therapy is increasingly used to enhance drug delivery and intensify the efficacy of topically applied drugs for scar treatment [45, 46]. Early intervention with PDL or fractional lasers (within weeks or months post injury) may be advantageous in mitigating scar contracture formation and trajectory with significant benefits in-patient rehabilitation, representing a potential breakthrough in the treatment of traumatic scars [23]. Surgical scars have been successfully treated using PDL, non-ablative fractional laser, or ablative fractional laser with positive results on the suture removal day or weeks post suture removal day, indicating intervention with laser and light might be applied as a safe and effective treatment to prevent traumatic scarring [31, 47, 48]. In another randomized, controlled, evaluator-blinded clinical trial, a single non-ablative fractional laser treatment at low to medium fluence performed 1 day prior or in the early phase of wound healing provided subtle but clinically detectable improvement, which indicates laser treatment may have the potential to optimize scar formation in full-thickness wound [49]. The authors recommend initiating the intervention with laser and light as early as possible to prevent the formation of traumatic scar base on the clinical experience and research study with the management of surgical sutures with laser and light (unpublished data).

Compared with full-field lasers, fractional laser therapy for traumatic scars is associated with a relatively low rate of complications [23]. Common adverse effects include transient erythema and localized swelling. Pinpoint bleeding and mild serious discharge might occur. Prolonged erythema, post-procedure pain requiring medications, scar exfoliation, infection, and transient post-inflammatory hyperpigmentation are rare in fractional laser treated traumatic scar patients. Nevertheless, the severe complications of new scarring and worsening of scarring were reported [50].

## Radiofrequency for the treatment of traumatic scars

Micro-plasma radiofrequency was developed as a minimally ablative technology. It utilizes the radiofrequency energy to provoke the nitrogen in the air to form a grid of high-energy foci called plasma sparks, which release heat as they return to the steady state and induce mild ablation of the epidermis and the formation of micro-channels of the dermis on scar tissue [51–53].

In recent years, combined with the fractional technology, micro-plasma radiofrequency has achieved good outcomes for the treatment of traumatic scars as well as acne scars [52, 54]. Comparable to the ablative fractional laser, the fractional micro-plasma radiofrequency technology (FMRT) has been emerging as a technology, characterizing with both ablation and thermal coagulation, which is capable to induce the neogenesis of collagen and remodeling of epidermis and dermis. Histological studies show that FMRT creates superficial and broad “crater”-like micro-channels, while the fractional carbon dioxide laser produces narrow and deep “cone”-like micro-channels [55]. Micro-plasma radiofrequency is also effective and safe for the treatment of post-burn hyperpigmentation, indicating its application for the treatment of traumatic scars in patients with Fitzpatrick skin type III or IV [51], whereas hyperpigmentation has been considered as a major adverse effect associated with laser treatment in those patients.

It is important to note that, although micro-plasma radiofrequency is believed to be a safe technology for traumatic scars therapy with minimal complications, it is critical to apply local cooling immediately post procedures as it is accompanied with local heat accumulation in treated tissues.

## Drug delivery and ultrasound for traumatic scar therapy

Global efforts have been put, reported in a myriad of studies, to clarify the mechanisms responsible for scar formation and emerging evidence has pointed to drug delivery of targeting molecule signaling as a promising avenue for scar therapy. An article has been published in which the relative studies on drug delivery for scar management were thoroughly reviewed and discussed [56]. Moreover, innovative technologies for drug delivery, such as transepidermal drug delivery (TED), provide an attractive alternative way to conventional injection with needle, which is painful and results in uneven distribution of medications. Both ablative fractional laser and micro-plasma radiofrequency have the capacity to create arrays of micro-channels on the scar, through which the therapeutic medications can be delivered into the deep layer of thick scar tissue efficiently and safely. Therefore, triamcinolone acetonide, 5-fluorouracil, collagenase, platelet-rich plasma, poly-l-lactic acid, and other medications or substances are often applied topically in the immediate postoperative period after fractional laser or micro-plasma treatment as the combination therapies for both hypertrophic and atrophic traumatic scars for better clinical outcomes [45, 46].

Recently, ultrasound technology has been incorporated to further facilitate the penetration of anti-scarring drugs into dermis through the micro-channels produced by micro-plasma radiofrequency. Ultrasound improves the delivery of therapeutic medications through mechanical (acoustic) pressure and torques by propagation of the ultrasound wave via the sonotrode to the distal horn and the creation of a “hammering” effect [57]. The combination therapy of micro-plasma radiofrequency with ultrasound was confirmed as an effective treatment method for both hypertrophic and atrophic scars with satisfactory results without the complications inherent in other methods [57–59]. The action mechanisms of micro-plasma radiofrequency or ablative fractional laser are completely different to ultrasound, and the combination can achieve a synergic action. Ultrasound technology aimed for drug delivery keeps on moving forward. It is worthwhile to mention that the combination of low-frequency ultrasound with advanced nanotechnology developed by Paithankar et al. [60] provides a very promising way for future traumatic scar therapy based on selective thermal effect.

## Conclusion

Laser, IPL, radiofrequency, and ultrasound-assisted medication delivery technologies alone or combined, have been proved to effectively and safely improve the appearance of the traumatic scars (thickness, texture, erythema, and pigmentation), decrease pain and itch, alleviate the contracture and improve function, and reduce the need for surgical excision. Both fractional lasers and radiofrequency devices can be applied solely for the treatment of both hypertrophic and atrophic traumatic scars. Vascular targeted devices PDL and IPL are solely or combined with fractional laser indicated for erythematous traumatic scars. Ultrasound is utilized as an adjuvant therapy with radiofrequency or fractional laser mainly for hypertrophic traumatic scars. Nevertheless, the cellular and molecular mechanisms responsible for the repairing and remodeling response to microscopic thermal injury introduced by fractional laser and radiofrequency therapies are elusive. Furthermore, treatment of traumatic scars with these technologies has not been optimized, and the efficacy and long-term outcome of these technologies have yet been compared until now. Therefore, both basic research to explore thoroughly the mechanisms, as well as randomized controlled clinical trials to explore the optimal treatment protocols, should be completed. The better understanding of these technologies will promote the appropriate implementation of these technologies in clinical practice for traumatic scars treatment.

## Abbreviations

CTGF: Connective tissue growth factor
Er:YAG: Erbium: yttrium-aluminum-garnet
Hsp: Heat shock protein
IPL: Intense pulsed light
PDGF: Platelet-derived growth factor
PDL: Pulsed dye laser
SMADs: Small mothers against decapentaplegic
TED: Transepidermal drug delivery
TGF-β: Transforming growth factor beta
VEGF: Vascular endothelial growth factor
